# Personalized allocation of acetylsalicylic acid therapy for secondary prevention of coronary artery disease

**DOI:** 10.3389/fcvm.2022.1004473

**Published:** 2022-09-27

**Authors:** Nischal N. Hegde, Navin Mathew, Rajesh Thachathodiyl, Jaideep C. Menon

**Affiliations:** Department of Cardiology, Amrita Institute of Medical Sciences, Kochi, India

**Keywords:** aspirin, coronary artery disease, aspirin resistance, aspirin regimen, secondary prevention ischemic heart disease, thromboxane B2

## Abstract

**Background:**

A single-daily dose of 75 mg of acetylsalicylic acid inhibits 100% of thromboxane-B2 synthesis within 30–60 min. Thromboxane-B2 synthesis then recovers slowly as new platelets are released from the bone marrow. Normally, only 10% of the platelets are replaced daily by new platelets entering circulation. Hence, 24 h after a dose of acetylsalicylic acid, thromboxane-B2 synthesis is still suppressed by more than 90%. Hence, there is an adequate anti-platelet effect even after 24 h of acetylsalicylic acid intake. However, some patients treated with once-daily acetylsalicylic acid may have an incomplete 24-h suppression of thromboxane-B2 synthesis due to increased platelet turnover. The response could be improved in such patients by twice-daily acetylsalicylic acid administration. This study aimed to identify such a group of patients who would benefit from a twice-daily dose of acetylsalicylic acid.

**Materials and methods:**

Serum thromboxane-B2 levels were measured in 79 patients with coronary artery disease receiving 75 mg of acetylsalicylic acid for secondary prophylaxis. Serum levels of thromboxane-B2 were measured after 4 and 24 h of acetylsalicylic acid intake. Patients were then classified into three groups: steady suppression group (serum thromboxane B2 is adequately suppressed at 4 and 24 h), i.e., adequate response to acetylsalicylic acid; fast recovery group (more than 10% rise in serum thromboxane-B2 levels at 24-h when compared to at 4-h) and non-responders (serum thromboxane-B2 levels of >3,100 pg/ml after 4 h of acetylsalicylic acid intake). Patients in the fast recovery group were given twice-daily acetylsalicylic acid and thromboxane-B2 levels were re-measured.

**Results:**

A total of 20 patients (24.3%) had steady suppression of thromboxane-B2 and 11 patients (13.9%) belonged to the fast recovery group, i.e., thromboxane-B2 levels were adequately suppressed at 4 h but had recovered by more than 10% at 24 h; which was adequately suppressed by twice-daily acetylsalicylic acid (p 0.004). A total of 48 patients (60.8%) were non-responders.

**Conclusion:**

Twice-daily acetylsalicylic acid may be beneficial if serum thromboxane-B2 levels at 4 h are <3,100 and >3,100 pg/ml at 24 h. If thromboxane-B2 levels at 4 and 24 h is <3100 pg/ml but if there is a >10% rise in serum thromboxane B2 at 24 h as compared to that at 4 h, then twice-daily acetylsalicylic acid should be considered. However, if thromboxane-B2 at 4 and 24 h is >3,100 pg/ml consider switching over to a P2Y12 inhibitor.

## Introduction

Acetylsalicylic acid 75–100 mg daily is recommended for secondary prophylaxis (1). It acts by irreversibly inhibiting nearly 100% of thromboxane B2 (TXB2) synthesis within 30–60 min (2). TXB2 levels then recover slowly as new platelets with non-acetylated COX-1 are released into circulation from the bone marrow. Since the platelet turnover rate is only about 10% per day, 24 h after a dose of acetylsalicylic acid, TXB2 levels also recover by only 10%. This has been the rationale for once-daily dosing of acetylsalicylic acid. When 90% of TXB2 synthesis is suppressed, thromboxane-mediated platelet aggregation is also maximally suppressed. Several studies reported that about a third of patients treated with acetylsalicylic acid showed inadequate 24-h suppression of TX synthesis (3). These patients were called poor responders (4). Even a small recovery of TXB2 synthesis above 10% can result in an increased risk of thrombosis (5). It is possible that TXB2 synthesis can be suppressed round the clock in such patients by twice daily acetylsalicylic acid administration. The 2021 ESC guideline recommends 75–100 mg of acetylsalicylic acid as a once daily dose in all patients for secondary prevention, irrespective of the individual’s acetylsalicylic acid responsiveness (1). Many of these patients suffer vascular events while on acetylsalicylic acid prophylaxis. On using Ticagrelor alone instead of acetylsalicylic acid for secondary prevention in patients with multivessel disease ticagrelor reduced the risk of MACE and coronary events, including coronary deaths but was associated with an increased risk of TIMI major bleed (6). In the COMPASS trial, it was demonstrated that adding rivaroxaban to acetylsalicylic acid decreased the number of vascular events. But adding rivaroxaban was associated with more major bleeding events when compared to acetylsalicylic acid alone (7). Moreover, replacing or adding a more expensive antithrombotic/anti-platelet has economic implications. Hence, it is important to identify the group of patients having inadequate TXB2 suppression with once-daily acetylsalicylic acid. These patients can be managed with twice-daily acetylsalicylic acid instead of the once-daily dose, thus significantly reducing further vascular events.

## Materials and methods

### Selection and description of participants

The study was carried out in a single center located in an institution in South India. This non-governmental institution is based on the not-for-profit model, and caters to a population of 30 million. The study group includes patients with coronary artery disease who were on single anti-platelet acetylsalicylic acid for secondary prophylaxis. Institutional ethical committee clearance (EC clearance number IRB-AIMS-2020-257) was obtained.

#### Inclusion criteria

1. Men and women aged ≥ 18 years.
2. Patients with coronary artery disease are already on a single anti-platelet acetylsalicylic acid (75 mg of enteric-coated acetylsalicylic acid) for secondary prevention.
3. On acetylsalicylic acid therapy for at least a 1-week duration.

#### Exclusion criteria

1. Use of drugs other than acetylsalicylic acid that interfere with cyclooxygenase (NSAIDs) within 1 week of study enrollment.
2. Use of concomitant P2Y12 inhibitors.
3. Platelet disorders (thrombocytosis/thrombocytopenia).
4. Other hematological disorders.
5. Inflammatory conditions as evidenced by an elevated CRP (>1 mg/dl).
6. On anti-coagulation therapy.
7. On acetylsalicylic acid therapy for ≤1 week.
8. Use of a dose other than 75 mg of acetylsalicylic acid.
9. Weight ≥ 70 kg.

#### Sample size

Based on the proportion of patients who have poor acetylsalicylic acid responsiveness (55%) due to enhanced platelet production observed in the earlier publication and with 20% allowable error and 95% confidence, the minimum sample size comes to 79 (8).

### Technical information

#### Primary objective

To investigate the frequency with which increased platelet production results in poor response to acetylsalicylic acid.

#### Secondary objective

To identify patient populations in whom increased platelet production is a major cause of poor response to acetylsalicylic acid. To determine the proportion of non-responders to single and split doses of acetylsalicylic acid.

#### Thromboxane B2 measurement

A total of 2 ml of blood samples were collected at 4 h (daily peak level) and 24 h after acetylsalicylic acid intake from patients who are meeting the inclusion and exclusion criteria. Samples were allowed to clot for 2 h at room temperature followed by centrifugation for 15 min at 1,000 × *g* at 2–8°C. The supernatant serum was stored at −40°C until assayed. Ebalscience Human TXB2 ELISA kit, with a coefficient of variation of <10%, was used. The sensitivity of the kit is 46.88 pg/ml and has a detection range of 78.13–5,000 pg/ml.

Frelinger AL, in their study involving 700 acetylsalicylic acid treated patients, concluded that patients with thromboxane B2 levels of >3,100 pg/ml had an increased incidence of MACE compared to those with thromboxane B2 levels of <3,100 pg/ml (9). In our study, we used this cut-off to define acetylsalicylic acid non-responsiveness.

**FIGURE 1:**
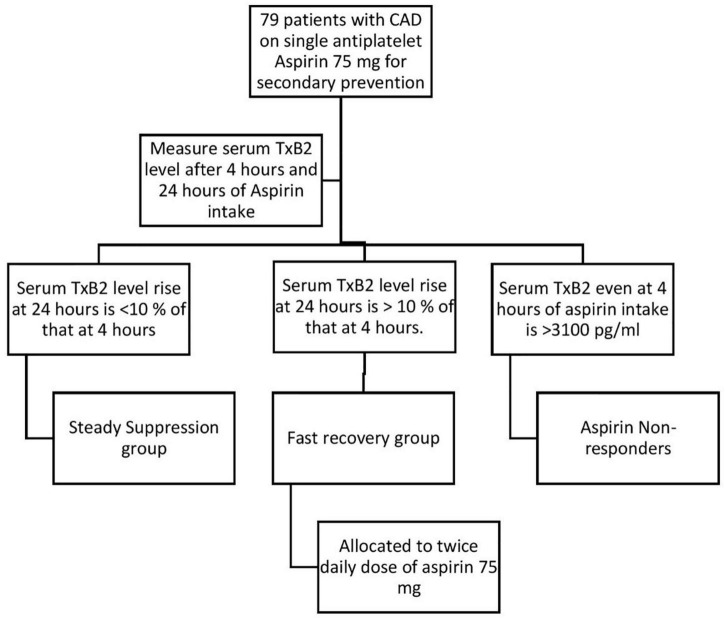
Study design. Abbreviations: CAD, coronary artery disease; TxB2, thromboxane B2.

Depending upon the results, the study population would be divided into three groups. First, the *steady suppression group* (TXB2 levels adequately suppressed at 4 and 24 h after acetylsalicylic acid intake), i.e., the group with an adequate response to acetylsalicylic acid. Second, the *fast recovery group* (TXB2 rise at 24 h is >10% of that at 4 h after acetylsalicylic acid intake). Third, the *non-responders* (No or only minimal TXB2 suppression, i.e., TXB2 level is more than the recommended 3,100 pg/ml). Patients in the fast recovery group are switched over to a twice daily regimen (Ecosprin 75 mg B.D. 8 a.m.–0–8 p.m.). TxB2 levels will be at re-measured after a minimum of 5 days of starting the twice-daily regimen (Figure 1).

### Statistics

Statistical analysis was performed using IBM SPSS version 22.0 software. Categorical variables are expressed using frequency and percentage. Continuous variables are presented by mean, SD, median, 25th, and 75th percentiles. To test the statistical significance of the changes in the median thromboxane B2 levels between 4 and 24 h of acetylsalicylic acid intake Wilcoxon Signed Rank test was used. To test the statistical significance of the difference in the median thromboxane B2 levels at 4 and 24 h between males and females, diabetics and non-diabetics, hypertensives and non-hypertensives, and dyslipidaemics and non-dyslipidaemics, Mann–Whitney *U* test was used. To test the statistical significance of the difference in the median thromboxane B2 levels at 4 and 24 h between three groups Kruskal–Wallis *H* test was used and multiple comparisons by Dunn’s Bonferonni test.

## Results

### Patient characteristics

A total of 79 patients were enrolled between September 2020 and August 2021. Table 1 presents patient characteristics. Their mean age was 67 ± 7.90 years, mean body weight was 60.77 ± 4.33 kilograms, and mean platelet count was 3.19 ± 0.551 lacs/μL. A total of 64 patients (81.01%) were males. A total of 52 patients (65.82%) had diabetes, 57 patients (72.15%) had hypertension, and 19 patients (24.05%) had renal dysfunction (eGFR < 60 ml/min/1.73 m^2^).

**TABLE 1:** Demographics.

Patient characteristics	Number of patients (Percentage)
**Sex**	
Male	64 (81.01%)
Female	15 (18.98%)
**Age (Years)**	
Average ± SD	67 ± 7.90
Minimum	52
Maximum	87
**Body weight (kilograms)**	
Average ± SD	60.77 ± 4.33
Minimum	51
Maximum	69
**Platelet count (lacs/μL)**	
Average ± SD	3.19 ± 0.55
Minimum	1.9
Maximum	4.4
Diabetes mellitus	52 (65.82%)
Hypertension	57 (72.15%)
Renal dysfunction	19 (24.05%)

### Mean thromboxane B2

The mean TxB2 levels after 24 h were significantly higher than at 4 h after acetylsalicylic acid intake (3414.961 ± 676.881 and 3147.388 ± 788.426 pg/ml, respectively, *p* < 0.001) (Table 2).

**TABLE 2:** Comparison of thromboxane B2 levels after 4 and 24 h of acetylsalicylic acid intake.

Time after acetylsalicylic acid intake	Thromboxane B2 (pg/ml)			
	Mean	Median	25th percentile	75th percentile
After 4 h	3147.388 ± 788.426	3,312	2,770	3,674
After 24 h	3414.962 ± 676.881	3,509	3,036	3,929
*p*-value	<0.001			

### Division into groups

In 20 patients (25.32%), the rise in TxB2 levels after 24 h of acetylsalicylic acid intake was <10% when compared to the TxB2 levels at 4 h of acetylsalicylic acid intake. These were the steady suppression group (group I). In 11 patients (13.92%), the rise in TxB2 levels after 24 h was >10% when compared to the TxB2 levels at 4 h of acetylsalicylic acid intake. These patients were labeled as fast recovery group (group II). A total of 48 patients (60.76%) had TxB2 levels of >3,100 pg/ml (inadequate TxB2 suppression) even at 4 h of acetylsalicylic acid intake. These patients were labeled as non-responders (group III) (Table 3).

**TABLE 3:** Division into groups.

Group	Number of cases	Percentage (%)
Steady suppression group (Group I)	20	25.32
Fast recovery group (Group II)	11	13.92
Non-responders (Group III)	48	60.76
Total	79	100

The mean thromboxane B2 levels in group I after 4 and 24 h of acetylsalicylic acid intake is 2661.511 ± 312.486 and 2885.833 ± 365.073 pg/ml, i.e., <10% rise after 24 h when compared to at 4 h. Whereas in group II the rise is >10% (1926.677 ± 863.356 and 2739.575 ± 983.116 pg/ml at 4 and 24 h, respectively, after acetylsalicylic acid intake). Even though group II had >10% rise in TxB2 levels after 24 h of acetylsalicylic acid intake when compared to at 4 h, the mean TxB2 levels at 24 h in group II were lower than in group I (2739.575 ± 983.116 and 2885.833 ± 365.073 pg/ml in group II and I, respectively).

The mean TxB2 levels after 4 h of acetylsalicylic acid intake in groups I and II are significantly lower than in group III (2661.511 ± 312.486, 1926.677 ± 863.356, and 3629.583 ± 383.248 pg/ml, respectively; *p* < 0.001 and <0.001) (Table 4).

**TABLE 4:** Comparing thromboxane B2 at 4 h between groups.

Pairs	*P*-value
Group I–Group II	0.2873
Group I–Group III	<0.001
Group II–Group III	<0.001

### Subgroup

There was no significant difference between mean TxB2 levels in males and females at 4 and 24 h after acetylsalicylic acid intake (3130.443 and 3219.689 pg/ml at 4 h, *p* = 0.348; 3417.864 and 3402.578 pg/ml at 24 h, *p* = 0.469).

There was no significant difference between mean TxB2 levels in diabetic and non-diabetic patients at both 4 and 24 h after acetylsalicylic acid intake (3222.736 and 3002.274 pg/ml at 4 h, *p* = 0.241; 3479.707 and 3290.268 pg/ml at 24 h, respectively, *p* = 0.241).

There was no significant difference in mean TxB2 levels in patients with and without renal disease at both 4 and 24 h of acetylsalicylic acid intake (3166.454 and 3141.351 pg/ml at 4 h, *p* = 0.905; 3444.182 and 3405.709 pg/ml at 24 h, respectively, *p* = 0.831).

There was no significant difference in TxB2 levels in patients with and without hypertension at both 4 and 24 h of acetylsalicylic acid intake (3193.917 and 3026.836 pg/ml at 4 h, *p* = 0.402; 3476.61 and 3255.236 pg/ml at 24 h, respectively, *p* = 0.194).

### Twice-daily acetylsalicylic acid

Patients in group II were given 75 mg of twice-daily acetylsalicylic acid. Mean TxB2 levels (measured after 24 h of taking the first dose of acetylsalicylic acid) were significantly lower (2739.575 ± 983.116 and 2134.749 ± 794.172 pg/ml before and after twice daily acetylsalicylic acid, respectively, *p* = 0.008).

## Discussion

In our study, 13.9% of the patients had enhanced platelet production causing reduced acetylsalicylic acid effectiveness. There was no statistically significant difference in the platelet turnover rate between males and females, diabetic and non-diabetics, those with and without renal disease, and with and without hypertension. A total of 60.8% of the patients were acetylsalicylic acid non-responders and 13.9% of the patients had adequate suppression of platelet activity on giving a split dose.

The meta-analysis of sixteen acetylsalicylic acid trials by the antithrombotic trialist collaboration group demonstrated an absolute reduction in adverse vascular events (6.7% per year in the acetylsalicylic acid group vs. 8.2% in controls, *p* < 0.0001) in patients-receiving acetylsalicylic acid for 2° prophylaxis (2). This forms the basis for the ESC class I recommendation of 75–100 mg of acetylsalicylic acid in all patients for secondary prevention. However, the one-dose-fits-all approach was questioned by Rothwell et al., who in a study demonstrated that only in patients weighing < 70 kg 75–100 mg dose of acetylsalicylic acid was effective. However, in patients weighing more than >70 kg, 80% of the men and 50% of the women, had no benefit (10). So, it was initially believed that 300 mg or more may be adequate for patients weighing more than 70 kg. However, this belief was short-lived when the results of the ADAPTABLE trial showed that in patients with established cardiovascular disease, taking 325 mg daily as compared to 81 mg, did not result in the reduction of vascular events (11). Many studies have demonstrated the superiority of multiple dosing as compared to a single daily dose of acetylsalicylic acid (12–14).

In our study, patients who were on single anti-platelet acetylsalicylic acid for secondary prophylaxis, had inadequate 24-h suppression of thromboxane B2 levels, with a 24-h thromboxane level being significantly higher than that at 4 h.

An ideal antiplatelet agent is one that would irreversibly inactivate a platelet protein (an enzyme or receptor) that cannot be resynthesized during a 24-h dosing interval, through a short-lived active moiety, thus limiting the extent and duration of any potential extraplatelet effect(s) (15). Acetylsalicylic acid is considered to be an ideal and economical antiplatelet. However, in our study, 13.92% of the patients had a significant (>10%) rise in TxB2 levels by end of 24 h after once-daily acetylsalicylic acid intake. This rise was suppressed by giving twice-daily acetylsalicylic acid. Hence, twice-daily acetylsalicylic acid might be able to achieve a better anti-thrombotic effect than once daily acetylsalicylic acid in the above group of patients.

In a study by Frelinger et al. in a cohort of 700 acetylsalicylic acid-treated coronary artery disease patients, MACE occurred more frequently in patients with high (>3,100 pg/ml) than in patients with low (<3,100 pg/ml) serum TXB2 levels (9). In the above 13.92% of the patients in our study (group II), even though >10% rise in TxB2 was found, the TxB2 levels even at 24 h were <3,100 pg/ml. But there is a linear relationship between TxB2 levels and thrombotic events (16). Therefore, twice-daily acetylsalicylic acid may be beneficial in this group of patients.

Surprisingly, 60.76% of our patients had serum TxB2 levels of >3,100 pg/ml even at 4 h after acetylsalicylic acid intake (group III). This lower-than-expected response to acetylsalicylic acid is often referred to as acetylsalicylic acid resistance (17). Hence, in this group of patients switching over to P2Y12 inhibitors may be more appropriate.

In our study, there was no statistically significant-difference in the mean TXB2 levels between males and females, diabetic and non-diabetics, with and without renal disease, and with and without hypertension at 4 and 24 h after acetylsalicylic acid intake. However, Rocca et al. and Spectre et al. reported that B.D. dosing of acetylsalicylic acid achieved more adequate thromboxane suppression in diabetics when compared to non-diabetics (18, 19).

In a study by Szczeklik et al., elevated thromboxane B2 level was associated with an increased risk of MACE in acute myocardial infarction patients (20). Further cardiovascular outcome studies are required comparing once daily with twice daily aspirin.

## Conclusion

**FIGURE 2:**
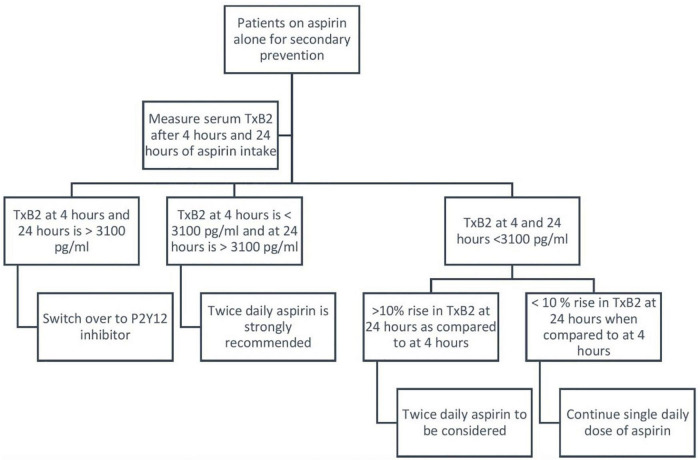
Suggested algorithm for personalized allocation of acetylsalicylic acid for secondary prevention of coronary artery disease. Abbreviation: TxB2, thromboxane B2.

One dose fits all may not be the right acetylsalicylic acid monotherapy strategy. Serum TxB2 measurements may be helpful in fine-tuning the acetylsalicylic acid regimen for individual patients. Twice-daily acetylsalicylic acid may be beneficial if TxB2 after 4 h of acetylsalicylic acid intake is <3,100 pg/ml and has increased to >3,100 pg/ml by 24 h. In cases where TxB2 at 4 and 24 h is <3,100 pg/ml but if there is a >10% rise in TxB2 at 24 h as compared to at 4 h then twice daily acetylsalicylic acid may be considered. If TxB2 at 4 and 24 h is >3,100 pg/ml consider switching over to a P2Y12 inhibitor (Figure 2).

## Limitations

This study has certain limitations. It is a single-center study with a small sample size. Depending upon the type of platelet function assay used, the incidence of acetylsalicylic acid resistance varies and there is a poor correlation among different assays.

## Data availability statement

The original contributions presented in this study are included in the article/supplementary material, further inquiries can be directed to the corresponding author.

## Ethics statement

The studies involving human participants were reviewed and approved by Amrita Institute of Medical Sciences (EC clearance number: IRB-AIMS-2020-257). The patients/participants provided their written informed consent to participate in this study.

## Author contributions

All authors listed have made a substantial, direct, and intellectual contribution to the work, and approved it for publication.
